# Seizure control in mono- and combination therapy in a cohort of patients with Idiopathic Generalized Epilepsy

**DOI:** 10.1038/s41598-022-16718-x

**Published:** 2022-07-19

**Authors:** Leonardo Zumerkorn Pipek, Henrique Zumerkorn Pipek, Luiz Henrique Martins Castro

**Affiliations:** 1 Faculdade de Medicina FMUSP, Universidade de Sao Paulo, Av. Dr. Arnaldo, 455 - Cerqueira César, São Paulo, SP 01246-903 Brazil; 2 Santa Casa de São Paulo School of Medical Sciences, São Paulo, Brazil; 3 Department of Neurology, Hospital das Clínicas HCFMUSP, Faculdade de Medicina, Universidade de Sao Paulo, São Paulo, SP Brazil

**Keywords:** Neuroscience, Epilepsy

## Abstract

Idiopathic Generalized Epilepsy (IGE) patients may not achieve optimal seizure control with monotherapy. Our goal was to evaluate the efficacy of combination therapy in a retrospective series of IGE patients receiving different antiseizure medication (ASM) regimens. We retrospectively identified all patients with adolescence onset IGE with typical clinical and EEG features from a single epilepsy specialist clinic from 2009 to 2020. We evaluated long-term seizure control, for VPA, LEV, LTG mono and combination therapy. We studied 59 patients. VPA was more commonly used in men (84%) than in women (44%) (*p* < 0.05). VPA was the initial drug of choice in 39% of patients, followed by LEV (22%) and LTG (14.9%). Thirty-nine patients (66.1%) achieved complete seizure control for at least one year. Fifty patients (84.7)% had partial control, without GTC occurrence, for at least one year. VPA was superior to LTG for complete seizure control (*p* = 0.03), but not for minor seizure control or pseudoresistance (*p* > 0.05). Combination therapy was superior to LEV and LTG monotherapy for complete control (*p* = 0.03), without differences for minor seizures and pseudoresistance outcomes (*p* > 0.05). Combination therapy not including VPA was also non-inferior to VPA monotherapy in all settings. Combination therapy was superior to LTG and LEV monotherapy in IGE, and may be equally effective including or not VPA. Combination therapy including LTG, LEV, and/or VPA is an effective treatment option after monotherapy failure with one of these ASM in IGE. Dual therapy with LEV–LTG should be considered in monotheraphy failure, to avoid fetal effects of in utero VPA exposure.

## Introduction

Idiopathic Generalized Epilepsy (IGE) affects 7.7 per 100,000 person/years in the United States^1^, and represents 15% to 20% of all epilepsies^2^. IGE is characterized by generalized tonic clonic (GTC), myoclonic, absence seizures, and typical EEG findings. Valproate (VPA) is the first-line antiseizure medication for IGE^3^. VPA use is limited by teratogenic effects. Seizure control in IGE for women with childbearing potential is usually worse, due to lower efficacy of other treatment options^3^. Lamotrigine (LTG) and Levetiracetam (LEV) monotherapies are not as effective as VPA, rendering up to 30% of IGE patients with unsatisfactory seizure control^4^.

Combination therapy for IGE, including or not VPA, has received less attention as a treatment option for IGE patients with incomplete seizure control^5^. Although used in clinical practice, effectiveness of combination therapy for IGE has not been specifically addressed. It is also unclear whether, after failure of an initial monotherapy for IGE, a second monotherapy should be the next regimen or if combination therapy would be a preferable option.

We performed a retrospective study to evaluate the efficacy of combination therapy in a series of patients with IGE.

## Materials and methods

### Study design

This was a single-center retrospective study of consecutive patients with Idiopathic Generalized Epilepsy (IGE) from January 2009 to June 2020. The study was conducted in accordance with the Declaration of Helsinki and International Council for Harmonization Good Clinical Practice guidelines. The institutional ethics committee (Hospital das Clínicas da Faculdade de Medicina da USP, São Paulo, Brazil) approved the study and waived the need for patient informed consent. (Protocol 44106121.0.0000.0068 Plataforma Brasil).

### Patient population

IGE patients were included if they had a history of adolescence onset of absences, myoclonic and/or generalized tonic–clonic seizures, and EEG findings consistent with a diagnosis of IGE, and if they were followed by a period of at least six months.

Patients were not included if they had focal seizures, EEG with more than 15% focal discharges, presence of radiological abnormalities that could explain seizures, or progressive neurologic dysfunction.

### Data collection

We collected data including (1) Patient demographics (age, gender, age at epilepsy onset, epilepsy duration, history of febrile seizures and family history of epilepsy, EEG, and Brain MRI), (2) Seizure type (GTC, myoclonic and absence seizures), precipitating events, frequency of seizures, and ILAE epilepsy syndrome (Juvenile Myoclonic Epilepsy—JME, Juvenile Absence Epilepsy—JAE and Generalized Tonic Clonic Seizures alone—GTCA), (3) Antiseizure medication use (ASM type, doses, duration of treatment, and plasma concentrations obtained for therapeutic drug monitoring, when available). We recorded the order of ASM medication for each patient (as the first, second, third, or nth treatment regimens). (4) Adverse events were defined as any untoward event attributed to an ASM, documented in the medical record, and classified as: severe, requiring hospitalization; moderate, requiring ASM discontinuation; mild, tolerable and not interfering with daily activities.

### Evaluation of efficacy

We defined three outcomes of seizure control for each treatment period, in terms of treatment efficacy for each patient: (1) seizure-free for all seizure types; (2) exclusive occurrence of minor non-disabling seizures (absence and/or myoclonic seizures); (3) pseudo-resistance (GTC occurrence exclusively with sleep deprivation, alcohol, illicit drug or irregular antiseizure medication use—nonadherence)^6^.

### Treatment period and outcomes

A treatment period was considered when a medication was used in an effective dose or if serum levels were in the therapeutic range. For complete seizure control, the treatment period required that the patient was seizure-free for at least six months. If the patient had a breakthrough seizure before six months, the treatment period was considered until the occurrence of the seizure. Treatment period duration was measured in months.

We evaluated the use of ASM for each period. ASM use was classified as monotherapy or combination therapy, and type of medication (valproate, levetiracetam, lamotrigine, topiramate, ethosuximide, and clobazam). All other drugs were classified as “other”.

### Statistical analysis

Estimates of mean, median, standard deviation, minimum and maximum values were calculated for quantitative variables. Qualitative variables were presented as absolute and relative frequencies. Association between qualitative variables was assessed with Pearson's chi-square or Fisher's exact test, according to expected values criteria (if 20% or more of the expected values are less than 5, then Fisher's exact test was used). Comparison of a quantitative variable between two independent groups was performed with Mann–Whitney test, and comparison between three independent groups with Kruskal–Wallis test, after testing normality using Kolmogorov–Smirnov test.

Time with total, partial, and pseudoresistance control of the disease was analyzed using the Kaplan Meier curve. Comparison for each medication was analyzed using Cox proportional hazards regression.

Patient data were divided into periods, according to medication use. If a patient did not present complete data in one treatment period, that period was excluded from analysis.

Significance level was 5%. In cases of multiple comparisons, the appropriate correction was applied.

## Results

### Study participants

A total of 1042 patients with medical records from a general neurology service from January 2009 to June 2020 were analyzed. Of these patients, 59 (5.7%) met study inclusion criteria. There were a total of 111 treatment periods, with a mean of 1.88 periods per patient.

All patients underwent EEGs in our service. Forty five (76.2%) displayed generalized spike wave or polyspike wave discharges, four (6.7%) showed rhythmic 3–4 Hz spike wave discharges, in the remaining ten patients, EEGs obtained in our service disclosed no epileptiform abnormalities, although all these patients had EEGs previously reported as generalized discharges. In six (10.1%) patients, focal discharges were seen (two frontal, two temporal, one frontotemporal and one occipital). In all cases focal discharges were not the predominant abnormal EEG pattern, and generalized discharges were also seen in all these patients. Demographic data for these patients are shown in Table 1.

**Table 1:** Patients’ demographic characteristics.

Demographic data	Patients
**Gender**	
Men	25 (42.4%)
Women	34 (57.6%)
Median age at symptom onset (years)	15 (IQR 5.75)
Median duration of follow-up (months)	59 (IQR 68.5)
**Seizure type**	
Generalized tonic clonic	56 (94.9%)
Myoclonic	33 (55.9%)
Absence	20 (33.9%)
Family history of epilepsy	37 (62.7%)
First degree	21 (35.6%)
Second degree	9 (15.2%)
Third degree	7 (11.9%)
History of febrile seizures	37 (62.7%)
**Current seizure control**	
Total control	33 (55.9%)
Minor seizures	9 (15.2%)
Pseudoresistance	8 (13.6%)
**EEG (in our service)**	
Generalized polyspike-wave	45 (76.2%)
3–4 Hz generalized spike wave	4 (6.7%)
Normal	10 (16.9%)

All patients, except one, had normal Brain MRI findings. That patient had a history of perinatal anoxia. For that patient, clinical presentation and EEG findings were consistent with IGE and not with a focal epilepsy.

The distribution of seizures type (absence, myoclonic, and generalized tonic–clonic) and ILAE epilepsy syndromes, according to gender, is shown in Table 2. There was no statistical difference regarding gender (*p* > 0.05).

**Table 2:** Seizure type classified by and ILAE IGE syndrome.

	Women*	Men*	Total
**ILAE classification**			
JAE	10 (29.4%)	4 (16%)	14 (23.7%)
JME	17 (50%)	16 (6.4%)	33 (55.9%)
GTCA	7 (20.6%)	5 (20%)	12 (20.3%)
**Seizure type**			
Absence	2 (5.9%)	1 (4%)	3 (5.1%)
Myoclonic	1 (2.9%)	3 (12%)	4 (6.8%)
GTC	7 (20.6%)	5 (20%)	12 (20.3%)
Absence + myoclonic	0%	0%	0 (0%)
Myoclonic + GTC	13 (38.2%)	10 (40%)	23 (39%)
Absence + GTC	8 (23.5%)	3 (12%)	11 (18.6%)
Absence + myoclonic + GTC	3 (8.8%)	3 (12%)	6 (10.2%)
Total	34 (100%)	25 (100%)	59 (100%)

*ILAE* International League Against Epilepsy, *JAE* Juvenile Absence Epilepsy, *JME* Juvenile Myoclonic Epilepsy, *CGTA* Generalized Tonic Clonic Alone, *GTC* Generalized Tonic Clonic.

*There was no significant difference based on gender for any seizure type prevalence (*p* > 0.05).

### ASM

**Figure 1:**
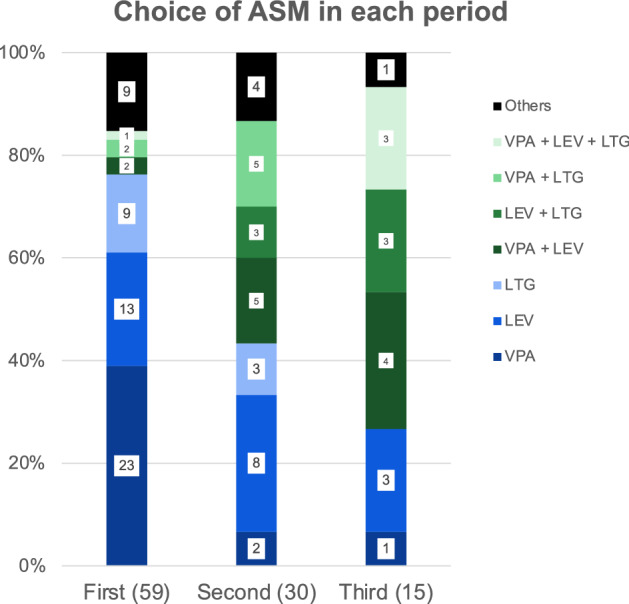
ASM choice in each period. Proportion of ASM used based on chronological periods. The absolute numbers of periods are presented in parentheses. Monotherapy regimens are represented in blue and combination therapy regimens in green. Other medications include mono or combination therapy with topiramate, phenobarbital, lacosamide, ethossuximide, and carbamazepine. The fourth period is not represented in this figure.

We analyzed 111 treatment periods (average of 37.8 months per period), classified chronologically as first, second, third, or fourth treatment periods. Figure 1 shows ASM used in each period. Combination therapy use was more common after each treatment period (*p* < 0.001).

Monotherapy with VPA, LEV, and LTG was used in 26, 24, and 12 periods, respectively. Combination therapy with VPA/LEV, VPA/LTG, LEV/LTG, and VPA/LEV/LTG was used in 11, 7, 6, and 4 periods, respectively. ASM daily dose ranges were: VPA—750 to 3500 mg, LEV—1000 to 3500 mg and LTG 200 to 700 mg. Doses for combination therapy (VPA/LTG) were lower due to drug interaction.

VPA use in mono or combination therapy was significantly more common in men, 88%, than in women, 44% (*p* = 0.002). There were no significant differences regarding any other ASM.

### Mono and combination therapy outcomes

Taking into account all periods for all ASM, 39 patients (66.1%) achieved complete seizure control for all types of seizures for at least one year. Fifty patients (84.7)% had partial control, without GTC occurrence, for at least one year.

**Figure 2:**
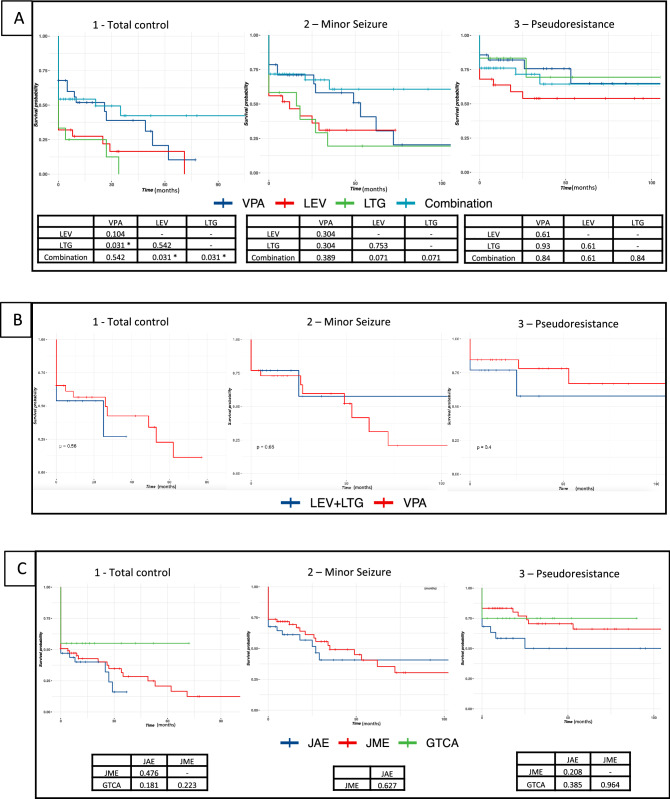
Outcomes for (**A**) Monotherapy and combination therapy; (**B**) VPA and LEV + LTG; (**C**) IGE syndromes. Survival curves for each level of control (total control, minor seizures, and pseudoresistance). The *p* values for pairwise comparisons are shown in each table below.

The Kaplan–Meier curves for each monotherapy and for all combination therapy are shown in Fig. 2A.

Regarding monotherapies, VPA was superior to LTG for complete seizure control (*p* = 0.031). We found no differences when considering outcomes for minor seizures or pseudoresistance (*p* > 0.05).

Combination therapy was superior to LEV and LTG monotherapy for total control (*p* = 0.031), but showed no difference for minor seizures and pseudoresistance outcomes (*p* > 0.05). Combination therapy was also noninferior to VPA monotherapy in all settings. Combination therapy showed favorable outcomes for longer periods compared to monotherapy, especially after four years, but this difference did not reach statistical significance.

We found no difference in outcome comparing combination therapy including or not VPA for all scenarios (*p* > 0.05). Combination therapy with LEV and LTG was non inferior to VPA monotherapy (Fig. 2B). There was also no statistically significant difference in outcomes when comparing ILAE IGE syndromes (Fig. 2C).

### Adverse effects, pseudoresistance and pregnancy

None of the patients had severe adverse effects requiring hospital admission. VPA therapy was discontinued in two cases due to concerns about weight gain (moderate side effects). Mild side effects that did not interfere with daily activities were present in 21 (18.9%) periods. The most common side effects were tremor and somnolence. LTG related mild skin rashes were seen in two patients. In both patients, LTG was re-introduced at a slower rate and doses, without rash recurrence. Combination therapy was not associated with a higher incidence of side effects.

There were 30 women of childbearing age in our cohort. Nine of them became pregnant during the study period. In one patient, VPA + LTG combination therapy was switched to LTG monotherapy due to concerns of risks associated with VPA in utero exposure. One patient had an unplanned pregnancy, VPA was maintained because VPA was the only drug that had controlled GTC seizures in that patient. The seven other patients that became pregnant were not on VPA.

The number of patients with myoclonus was similar with all ASM (*p* > 0.05).

We had 19 out of 111 (17.1%) outcomes of pseudoresistance. In 18 of them, patients were on monotherapy and one patient was on combination therapy (Table 3).

**Table 3:** Pseudoresistance effects: ASM use.

ASM	Number of events of pseudoresistance n/N
VPA	7/26 (26.9%)
LEV	6/24 (25%)
LTG	5/12 (41.7%)
VPA + LEV	1/11 (9.1%)

*ASM* antiseizure medication, *n* number of pseudoresistance events, *N* number of patients on the ASM.

## Discussion

In this retrospective study of a consecutive series of 59 IGE patients evaluating 111 treatment periods, we found that combination therapy was superior to LEV and LTG monotherapies, and combination therapy, not including VPA, was non-inferior to VPA monotherapy. Complete seizure control was seen in less than 50% of patients in a five-year follow-up.

Demographic features of the population in this study were similar to other studies that evaluated adolescence onset IGE. The mean age of onset of seizures in IGE was between 14 and 15 in other studies. Seizure types seen in our sample are similar to previously reported data (90% TGC, 20% absence and 100% myoclonic seizures^7–12^), except for a smaller proportion of 55.9% patients presenting myoclonic seizures. Eighty percent of patients in our study presented with myoclonic seizures and/or absences, associated or not with GTC. Only 20% of patients presented exclusively with GTC seizures, and all these them had awake and sleep EEG findings with generalized epileptiform discharges to support the diagnosis of IGE. A family history of epilepsy was present in our cohort in 35.6% of first-degree family members, similar to previously reported studies^9,12–14^. Women were slightly overrepresented in our study (57.6% of cases).

This study reflects therapeutic strategies used in routine clinical practice and may, therefore, more likely replicate ASM efficacy in a real-world scenario. Additionally, different outcomes and levels of control were considered, where both complete seizure control, as well as occurrence of minor non-disabling seizures, were considered as acceptable outcomes, reflecting usual clinical practice.

We also evaluated pseudoresistance to separate patients with real drug resistance from all other causes of seizure breakthrough. There is no clear definition for “pseudoresistance”, but it is commonly referred to as “inadequate lifestyle”, which includes non-adherence, sleep deprivation and alcohol^6^*.* It is difficult to tease apart different causes for pseudoresistance since multiple triggers often occur concomitantly. In our study, almost all patients classified as pseudoresistance due to “non-adherence” also presented other concomitant seizure triggers. For that reason, we did not separate a specific cause for non-adherence. Non-adherence was infrequent in our cohort.

Our median follow-up period was 59 months, considerably longer compared to previous studies of ASM efficacy in IGE, allowing evaluation of long-term efficacy for each treatment regimen. Additionally, we evaluated different treatment regimens periods for the same patient (mean 1.88), allowing evaluation of real-life efficacy of each regimen. Similar studies that also analyzed combination therapy in IGE^5,15^ used a prospective approach, evaluating efficacy of a single drug as an add-on therapy. In these studies, treatment time ranged from 16 to 36 weeks, including an uptitration period. The sample size was around 120, half of them in the placebo group.(Supplement 1)

Although IGE is commonly regarded as an ASM responsive epilepsy, long-term optimal seizure control remains a challenge. A significant proportion of patients have uncontrolled seizures^4,16^. Although newer ASM show efficacy for IGE seizure types^17^, valproate remains the first option for men and women without childbearing potential^3^. In our cohort, almost 40% received VPA monotherapy as their initial treatment. Valproate use is preferably avoided in women with childbearing potential^3^. In our sample, valproate use was twice more common in men than in women, both in mono- and combination therapy. Since the Standard and New Antiepileptic Drugs (SANAD) Study^18^, valproate have been considered the drug of choice for patients with IGE. The ILAE treatment protocol published in 2022^19^ also recommends valproate as the most effective drug to initiate treatment. For women of childbearing age, risks must be weighed against benefits of treatment.

Our findings indicate that VPA is superior to LTG regarding complete seizure control in monotherapy, underscoring that VPA remains a key therapeutic option for IGE, despite potential teratogenicity. We did not find significant differences in outcome considering occurrence of minor seizures as adequate seizure control. We also did not find any difference between LEV and LTG efficacy in monotherapy. Previous studies have provided data indicating LEV superiority^20^, or a similar effect, compared to lamotrigine^21^. Alternative treatments, using lamotrigine and levetiracetam in monotherapy, show inferior results, and patients can be resistant to all three drugs in monotherapy^22^.

Furthermore, we did not find any difference in efficacy of different medication regimens and pseudoresistance, which is not an unexpected finding, since pseudoresistance is more likely associated with lifestyle issues. Due to small numbers, we were not able to determine that any antiseizure medication regimen had a greater propensity to non-adherence.

Combination therapy is an alternative when monotherapy fails, but studies evaluating combination therapies in IGE are still scarce^5,15^. In placebo-controlled add-on protocols, LEV^5^ or LTG^15^ showed efficacy in IGE, with only mild side effects. These studies did not compare different ASM combinations of drugs, not allowing a comparison between different combination therapies. Another study^23^ evaluating drug-resistant juvenile myoclonic epilepsy patients suggested that the best combination therapy is valproate and lamotrigine.

Valproate and lamotrigine combination therapy was considered the best combination therapy in a retrospective study. An expert review^24^ on the diagnostic and therapeutic approach to drug-resistant juvenile myoclonic epilepsy did not evaluate the efficacy of combination therapy.

In our sample, combination therapies with LTG, LEV, and VPA were used more frequently in the second and third treatment periods, ranging from 8.4% (5/59) of the initial treatment to 43.3% (13/30) of the second treatments, to 66.6% (10/15) of the third treatments. On survival curve analysis, combination therapy was superior to LEV and LTG monotherapies, and non-inferior to VPA monotherapy. Patients that do not achieve adequate seizure control with these medications in monotherapy, may benefit from combination therapy. Combination therapy appears to retain efficacy for longer periods, but our sample size was not powered to achieve statistical significance. In our study, combination therapy was individualized for each patient, and involved different ASM choices. We found no difference in efficacy, whether combination therapy included VPA or not, indicating that LEV–LTG may be used in patients in whom VPA should be avoided^22^.

Although this study evaluated a relatively small sample size, study design allowed analysis of different treatment periods for each patient. Number of patients analyzed in second, third and fourth treatment trials was progressively smaller, since most patients responded to the initial monotherapy treatments. Only 32 combination therapy periods were evaluated. Despite being used in more ASM resistant patients, seizure control was still possible.

A considerable portion of patients had seizures triggered either by sleep deprivation or lack of treatment adherence, underscoring the importance of lifestyle changes and strict treatment adherence to achieve optimal seizure control.

This study analyzed data from a single practice from an epilepsy specialist database, and some of the patients had previously been diagnosed and treated by other neurologists.

This study design has some advantages, as patients’ follow-up was longer compared to other studies, and reflects usual clinical practice, with close patient-physician communication, with an effective report of breakthrough seizures and side effects.

Future studies should evaluate the efficacy of combination therapy with LEV and LTG (and other broad-spectrum ASM) in ASM resistant IGE, and should also evaluate if combination therapy, rather than a second or third monotherapy would be more effective in achieving seizure control in this patient population. Moreover, considering that non-adherence and other causes of seizure triggers are relatively common, analysis of different ASM under those conditions is important for the clinical practice.

## Conclusion

VPA remains the first treatment option in IGE cases without contraindications to its use. Combination therapy was superior to LTG and LEV monotherapy in IGE and may be equally effective when it includes VPA. Combination therapy including LTG, LEV and/or VPA is a reasonable treatment option after monotherapy failure with one of these ASM in IGE. Dual therapy with LEV–LTG should be considered when needed to avoid VPA in utero exposure effects.

## Supplementary Information

Supplementary Information.

## Data Availability

The datasets used and/or analysed during the current study are available from the corresponding author on reasonable request.
