# Supplementary material for: Seizure control in mono- and combination therapy in a cohort of patients with Idiopathic Generalized Epilepsy
Source: Sci Rep. 2022 Jul 19;12:12350. doi: 10.1038/s41598-022-16718-x (PMC9296520; doi:10.1038/s41598-022-16718-x)
Supplement: Supplementary file 1 — Supplementary Information. [file 41598_2022_16718_MOESM1_ESM.docx]

**Supplement 1:** Comparison of main studies for combination therapy

|  | Pipek et al. | Rosenfeld et al. | Biton et al. |
| --- | --- | --- | --- |
| Sample size | 59 divided in 111 treatment periods | 240 (112 treatment, 128 placebo) | 117 (58 LTG, 59 placebo) |
| Study design | Retrospective cohort | RCT | RCT |
| Combination therapy | VPA, LEV and LTG with combinations | LEV as add on therapy to either VPA, LTG, carbamazepine or topiramate | LTG as add on therapy to either VPA, phenytoin, phenobarbital, carbamazepine or primidone |
| Seizure type | Myoclonic, absence and GTC | Myoclonic and GTC | Myoclonic, absence and GTC |
| Period evaluated | 36 – 1596 weeks (median of 354 weeks) | 16—24 weeks | 12-weeks (after uptitration) |
| Conclusion | Combination therapy was superior to LEV and LTG monotherapy for complete control (p = 0.03), without differences for minor seizures and pseudoresistance outcomes (p > 0.05). Combination therapy was also non-inferior to VPA monotherapy in all settings. | Response rates with levetiracetam as add-ons on therapy was better than placebo (61.0% vs. 24.7%) | 72% of lamotrigine-treated patients compared with 49% of placebo-treated patients experienced a > or = 50% reduction in frequency of PGTC seizures |
